# The ABC Transporter *Eato* Promotes Cell Clearance in the *Drosophila melanogaster* Ovary

**DOI:** 10.1534/g3.117.300427

**Published:** 2018-01-02

**Authors:** Clarissa S. Santoso, Tracy L. Meehan, Jeanne S. Peterson, Tiara M. Cedano, Christopher V. Turlo, Kimberly McCall

**Affiliations:** * Department of Biology, Boston University, Massachusetts 02215; † Program in Molecular Biology, Cell Biology, and Biochemistry, Boston University, Massachusetts 02215

**Keywords:** cell death, engulfment, ABC transporter, *ced-7*, *Eato*

## Abstract

The clearance of dead cells is a fundamental process in the maintenance of tissue homeostasis. Genetic studies in *Drosophila melanogaster*, *Caenorhabditis elegans*, and mammals have identified two evolutionarily conserved signaling pathways that act redundantly to regulate this engulfment process: the *ced-1/-6/-7* and *ced-2/-5/-12* pathways. Of these engulfment genes, only the *ced-7/ABCA1* ortholog remains to be identified in *D. melanogaster*. Homology searches have revealed a family of putative *ced-7/ABCA1* homologs encoding ATP-binding cassette (ABC) transporters in *D. melanogaster*. To determine which of these genes functions similarly to *ced-7/ABCA1*, we analyzed mutants for engulfment phenotypes in oogenesis, during which nurse cells (NCs) in each egg chamber undergo programmed cell death (PCD) and are removed by neighboring phagocytic follicle cells (FCs). Our genetic analyses indicate that one of the ABC transporter genes, which we have named *Eato* (Engulfment ABC Transporter in the ovary), is required for NC clearance in the ovary and acts in the same pathways as *drpr*, the *ced-1* ortholog, and in parallel to *Ced-12* in the FCs. Additionally, we show that *Eato* acts in the FCs to promote accumulation of the transmembrane receptor Drpr, and promote membrane extensions around the NCs for their clearance. Since ABCA class transporters, such as CED-7 and ABCA1, are known to be involved in lipid trafficking, we propose that Eato acts to transport membrane material to the growing phagocytic cup for cell corpse clearance. Our work presented here identifies *Eato* as the *ced-7/ABCA1* ortholog in *D. melanogaster*, and demonstrates a role for *Eato* in Drpr accumulation and phagocytic membrane extensions during NC clearance in the ovary.

PCD is a fundamental biological process in animal development and tissue homeostasis. Cells undergoing PCD are selectively cleared by phagocytes in a multi-step engulfment process involving recognition followed by internalization of the dying cell (Arandjelovic and Ravichandran 2015; Green *et al.* 2016). In some instances, phagocytes can promote the death of their target cells (Reddien *et al.* 2001; Brown and Neher 2012; Timmons *et al.* 2016). Abnormal regulation of the engulfment process has been implicated in several human diseases, including developmental malformations, physiological disorders, autoimmunity, neurodegeneration, and cancer (Arandjelovic and Ravichandran 2015; Green *et al.* 2016).

Engulfment is generally performed by “professional” phagocytes, such as mammalian macrophages, whose primary function is the phagocytosis of cellular debris. In tissues where professional phagocytes have little to no access, resident cells can function as “nonprofessional” phagocytes to remove dead cells (Arandjelovic and Ravichandran 2015; Green *et al.* 2016). For example, in the *Drosophila* ovary, a system closed to circulating cells, clearance of dying NCs is accomplished by neighboring epithelial cells called FCs (Giorgi and Deri 1976; Etchegaray *et al.* 2012). Current evidence suggests that engulfment by professional and nonprofessional phagocytes is regulated similarly (Arandjelovic and Ravichandran 2015; Green *et al.* 2016).

Extensive genetic studies in *Caenorhabditis elegans* have identified two parallel but partially redundant signaling pathways, CED-1/-6/-7 and CED-2/-5/-12, which regulate the engulfment process (Ellis *et al.* 1991; Kinchen *et al.* 2005). These pathways appear to be conserved in mammals as MEGF10/GULP/ABCA1 and Crk/DOCK180/ELMO, and in *Drosophila melanogaster* as Drpr/Ced-6 and Crk/Myoblast city/Ced-12, respectively (Mangahas and Zhou 2005). However, the *D. melanogaster* ortholog for CED-7/ABCA1 has not been identified.

The genes *ced-7* and *ABCA1* encode members of the ABCA subfamily of ABC transporters (Luciani and Chiminil 1996; Wu and Horvitz 1998). ABC transporters are important in a wide range of physiological processes and can translocate a variety of substrates, including sugars, ions, lipids, and proteins (Rees *et al.* 2009; ter Beek *et al.* 2014; Wilkens 2015). Mutations that abolish the ATP-binding function of CED-7 or ABCA1 cause engulfment defects that lead to the accumulation of cell corpses *in vivo* (Luciani and Chiminil 1996; Wu and Horvitz 1998; Hamon *et al.* 2000).

In *C. elegans*, CED-7 has been shown to be required in both the phagocytic and the dying cell for efficient engulfment (Wu and Horvitz 1998). In mammals, ABCA1 is clearly required in phagocytic cells (Hamon *et al.* 2000), but whether the protein is required in dying cells *in vivo* has not been determined. *In vitro* studies in mouse cell culture hemocytes and thymocytes have demonstrated a role for ABCA1 in phosphatidylserine (PtdSer) exposure following apoptotic stimuli (Hamon *et al.* 2000), suggesting that ABCA1 may act in dying cells to promote cell corpse recognition. In contrast, PtdSer was clearly detected on the surface of cell corpses *in vivo* in *ced-7* mutants (Mapes *et al.* 2012), indicating that CED-7 is not required for PtdSer exposure in *C. elegans*.

Multiple reports have speculated whether CED-7/ABCA1 acts as a lipid transporter. Indeed, most ABCA-type transporters appear to be involved in lipid trafficking (Vasiliou *et al.* 2009; Quazi and Molday 2011). In mammals, ABCA1 has been shown to promote the transport of lipids from the Golgi to the plasma membrane, and the efflux of lipids to form high-density lipoproteins (HDLs) (Hamon *et al.* 2000; Orsó *et al.* 2000). In humans, deficiency for *ABCA1* is implicated in Tangier disease, a recessive disorder of lipid metabolism characterized by the lack of HDLs due to defective translocation of membrane lipids (Hamon *et al.* 2000; Orsó *et al.* 2000; Vasiliou *et al.* 2009).

In *C. elegans*, CED-7 has been observed to play a role in both intracellular and extracellular lipid trafficking during engulfment. CED-7 was shown to act with CED-1, CED-6, and DYN-1 to promote the intracellular delivery of vesicles to the phagocytic cup, presumably to provide lipid and protein materials to the growing membrane for pseudopod extensions (Yu *et al.* 2006). CED-7 has also been shown to be required for the presence of extracellular vesicles and is proposed to mediate the exocytosis of vesicles containing engulfment signals, such as the bridging molecule TTR-52, which facilitates CED-1 recognition of PtdSer (Mapes *et al.* 2012). However, because CED-7 activity appears to be required in both the phagocytic and dying cells for engulfment in *C. elegans* (Wu and Horvitz 1998), it has been complicated to determine exactly where CED-7 acts in the signaling pathway.

Downstream of CED-7/ABCA1, a prominent feature observed during engulfment, is the clustering of the transmembrane receptors CED-1/MEGF10 at the phagocytic cup (Zhou *et al.* 2001). *In vivo* studies in *C. elegans* and *in vitro* studies in mouse cell culture, respectively, show that CED-1/MEGF10 clusters around the cell corpse and facilitates cell clearance in a manner dependent on CED-7/ABCA1 (Zhou *et al.* 2001; Hamon *et al.* 2006). Given its putative role in lipid transport, it is tempting to speculate that CED-7/ABCA1 may function at the phagocytic cup to remodel the local lipid composition, and perhaps generate domains such as lipid rafts to which CED-1/MEGF10 can be recruited.

To identify and characterize the CED-7/ABCA1 ortholog in *D. melanogaster*, we used the *D. melanogaster* ovary as an *in vivo* model system to study cell death and engulfment. Two distinct germline PCD events, developmental PCD in late oogenesis and stress-induced PCD in midoogenesis, have been well characterized in the ovary (Jenkins *et al.* 2013; Peterson *et al.* 2015). The ovary is comprised of a bundle of 15–20 ovarioles, sheaths of progressively developing egg chambers through 14 stages of oogenesis. Each egg chamber contains 16 interconnected germline-derived cells, composed of a single oocyte and 15 NCs, surrounded by a layer of somatically-derived FCs (King 1970; Spradling. 1993). As each oocyte reaches maturation, the 15 NCs undergo PCD and are cleared by the neighboring FCs. We have found that phagocytosis genes including *drpr* and *Ced-12* are required for NC clearance, as their loss-of-function results in stage 14 egg chambers that exhibit persisting NC nuclei (PN) (Timmons *et al.* 2016). Additionally, genetically inducing death in a small subset of the phagocytic FCs inhibits the death and removal of the NCs, suggesting that the FCs nonautonomously promote the death and removal of the NCs via phagoptosis.

During midoogenesis, PCD of the germline can occur in response to stress, such as protein starvation. PCD in midoogenesis requires active caspases, including death caspase 1 (Dcp-1), and autophagy genes, suggesting that death is executed via apoptotic and autophagic cell death pathways (Jenkins *et al.* 2013). As the NCs degenerate, the surrounding FCs synchronously enlarge to engulf the germline debris. Similar to clearance in late oogenesis, this process in midoogenesis is regulated by *drpr* and *Ced-12* (Etchegaray *et al.* 2012; Meehan *et al.* 2015a). *drpr* and *Ced-12* mutants produce egg chambers that exhibit dying NCs, with FCs that fail to enlarge or take up the germline material.

The engulfment pathways first defined in *C. elegans* are highly conserved in *D. melanogaster*, but a *ced-7/ABCA1* ortholog has not been reported. The *D. melanogaster* genome contains 56 ABC genes, of which 10 encode ABCA type transporters similar to CED-7/ABCA1. Only two of the ABCA genes, *CG31731* and *CG1718*, are expressed at appreciable levels in the ovary (FlyBase). We functionally analyzed these two genes in the *D. melanogaster* ovary and found that *CG31731* mutants show profound defects in NC clearance. Moreover, *CG31731* appears to play a similar role to *ced-7/ABCA1* in the engulfment process. Thus, *CG31731* likely serves a CED-7/ABCA1 role in engulfment in the *D. melanogaster* ovary, and hereafter will be referred to as *Eato*.

## Materials and Methods

### Fly strains and manipulations

All stocks were obtained from the Harvard Transgenic RNAi Project (Perkins *et al.* 2015) (*CG31731^HMC06027^*, *CG1718^HMS01821^*, *CG1718^HMS01796^*, and *Ced-12^HM05042^*), the Bloomington *Drosophila* Stock Center (BDSC) (*CG31731^MI14571^*, *CG31731^f02254^*, and *CG1718^mir-1007-KO^*), or the Vienna *Drosophila* Resource Center (*CG31731^GD1133^*, *CG31731^KK104197^*, *CG1718^GD3708^*, and *CG1718^KK100452^*), with the exception of the *drpr*^Δ^*^5/^*^Δ^*^5^* strain (Freeman *et al.* 2003) provided by Estee Kurant.

The initial *Eato^Mi^/CyO* strain received from the BDSC was homozygous lethal, but *Eato^Mi^/Df(2L)BSC812* flies were viable, so we used the *Df(2L)BSC812* strain (which uncovers *Eato*) to generate the homozygous *Eato^Mi/Mi^* strain. Specifically, we crossed *Eato^Mi^/CyO* with *Df(2L)BSC812/CyO*, and the *Eato^Mi^/Df(2L)BSC812* F1 progeny exhibiting straight wings were mated to allow background lethal mutations to recombine off. Since *Df(2L)BSC812* is marked by a *w^+^* marker while the *Eato^Mi^* construct carries no eye pigment marker, F2 progeny exhibiting white eyes were collected to select against the deficiency chromosome and generate the *Eato^Mi/Mi^* strain.

To make germline clones, we generated an *Eato^Mi^* FRT 40A stock by recombination and used the *FLP ovo^D^* system (Chou and Perrimon 1996). RNAi knockdown lines were generated using the GAL4-UAS binary system, with *GAL4* expressed under control of an endogenous tissue-specific enhancer, specifically *GR1*, which is expressed in all FCs after stage 3 including the stretch FCs (Goentoro *et al.* 2006), and *nanos*, which is expressed in the NCs (Rørth 1998).

All strains were reared on standard cornmeal molasses yeast media at 25°. Prior to dissection, adult males and females were transferred to a vial containing fresh media and a teaspoon of yeast paste, and conditioned for ∼2 d. To induce cell death in midstage egg chambers, adults were conditioned with yeast paste for ∼1 d then transferred to apple juice agar vials and starved of yeast for the last 16–20 hr period prior to dissection.

### Staining and microscopy

Ovaries were dissected in Grace’s Insect Media (Fisher) and then processed as previously described (Meehan *et al.* 2015b). Primary antibodies used were: α-Drpr [1:50; Developmental Studies Hybridoma Bank (DSHB)], α-Dlg (1:100; DSHB), and α-cleaved Dcp1 (1:100; Cell Signaling). Secondary antibodies used were: goat-α-rabbit Cy3, goat-α-mouse Cy3, and goat-α-rabbit Alexa Fluor 647 (1:200; Jackson ImmunoResearch). Ovaries were mounted in Vectashield with 4′,6-diamidino-2-phenylindole dihydrochloride (DAPI) (Vector Laboratories) and slides were stored at 4°. Egg chambers were imaged on an Olympus BX60 upright fluorescence microscope or an Olympus FV10i confocal microscope, and images were processed in ImageJ.

### Quantitative RT-PCR

RNA samples were extracted from pooled ovaries using the QIAGEN RNeasy Mini Kit, and then converted to cDNA using the Thermo Scientific Maxima First Stand cDNA Synthesis Kit. qPCR was performed following the Promega GoTaq qPCR Master Mix protocol with two primer sets, one flanking the fourth and fifth exons and another flanking the 14th and 15th exons of *Eato*. The results were normalized to *Rpl32* as an internal control.

### Quantifications

To quantify engulfment, the number of PN in each stage 14 egg chamber was counted. The criteria for a stage 14 egg chamber was fully developed dorsal appendages (Jia *et al.* 2016). The egg chambers were then grouped into bins of 0 PN, 1–3 PN, 4–6 PN, 7–9 PN, 10–12 PN, or 13–15 PN, and each bin was presented as a percentage of all stage 14 egg chambers quantified per genotype. Alternatively, the average number of PN in stage 14 egg chambers from each genotype was presented. “*n*” represents the total number of stage 14 egg chambers quantified.

To quantify Drpr accumulation or stretch FC (SFC) membrane extensions around the NCs, using ImageJ, the length around each NC nucleus that was α-Drpr- or GFP-positive was measured as a percentage of the circumference around each NC nucleus. Each NC nucleus was then grouped into bins of 0–10, 11–30, 31–50, 51–70, 71–90, or 91–100%, surrounded by α-Drpr or GFP, and each bin was presented as a percentage of all NC nuclei quantified per genotype. “*n*” represents the total number of NC nuclei quantified.

All quantifications were performed blind and statistical analyses were performed in Graphpad Prism.

### Data availability

All strains and reagents are available upon request.

## Results

### Eato encodes an ABC transporter similar to ced-7/ABCA1

**Figure 1:**
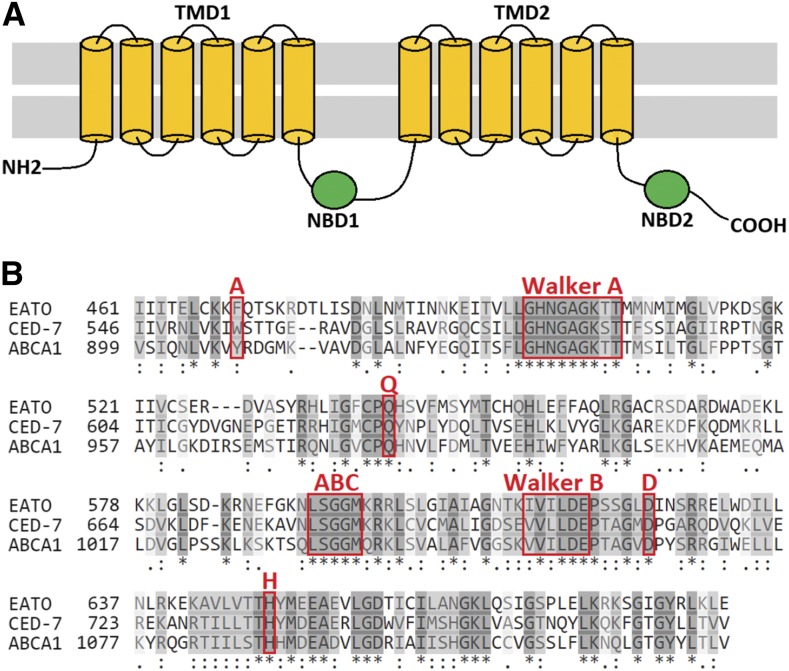
*CG31731*/*Eato* encodes an ATP-binding cassette (ABC) transporter. (A) Schematic of a general ABC transporter, with two transmembrane domains (TMDs) comprised of 12 transmembrane segments and two nucleotide-binding domains (NBDs). (B) The predicted NBD1 amino acid sequences of *Eato*, *ced-7*, and *ABCA1* aligned using CLUSTALO provided by UniProt. The similarities (light gray) and identities (dark gray) are highlighted. The conserved A-loop, Walker A motif, Q- loop, ABC signature motif, Walker B motif, D-loop, and H-loop, are indicated.

A search of databases [FlyBase, UniProt, and the *Drosophila* RNAi Screening Center Interactive Ortholog Prediction Tool (DIOPT)] revealed that the predicted amino acid sequence of *Eato* encodes an ABC transporter of the ABCA subfamily, which includes CED-7 and ABCA1. Like other ABC transporters, *Eato* encodes a protein with two transmembrane domains (TMDs) and two cytosolic nucleotide-binding domains (NBDs) (Figure 1A) with predicted ATP-binding and catalytic capability. Each NBD was found to contain an “A-loop” (aromatic), “Walker A motif” (GxxGxGKS/T), “Q-loop” (glutamine, Q), “ABC signature motif” (L/YSGGQ/M), “Walker B motif” (φφφφDE), “D-loop” (aspartate, D), and “H-loop” (histidine, H), in highly conserved sequential and spatial organization (Figure 1B), classifying the protein as an ABC transporter (Rees *et al.* 2009; ter Beek *et al.* 2014). Additional structural analyses of the predicted amino acid sequence indicated that each Eato TMD contains six hydrophobic α-helical segments, generating a 12-pass transporter. In comparison, both CED-7 and ABCA1 each contain 15 transmembrane segments.

Using Basic Local Alignment Search Tool (BLAST) algorithms to compare the predicted amino acid sequences of Eato with that of CED-7 and ABCA1, a substantial amount of similarity and identity were found throughout their entire length, most notably in the catalytic NBD regions. Overall, Eato was found to be 20% identical to CED-7 and 18% identical to ABCA1. More specifically, the NBDs of Eato were found to be 35% identical and 36% similar to those of CED-7, and 40% identical and 36% similar to those of ABCA1. Additional homology searches using the DIOPT (Hu *et al.* 2011) revealed that *Eato* is the best predicted ortholog of *ced-7* and also a predicted ortholog of *ABCA1*. Alignment of the proteins provided by DIOPT revealed a 24% identity and 42% similarity overall between Eato and CED-7, and a 26% identity and 43% similarity between Eato and ABCA1.

### Eato mutants have persisting NC corpses in the ovary

**Figure 2:**
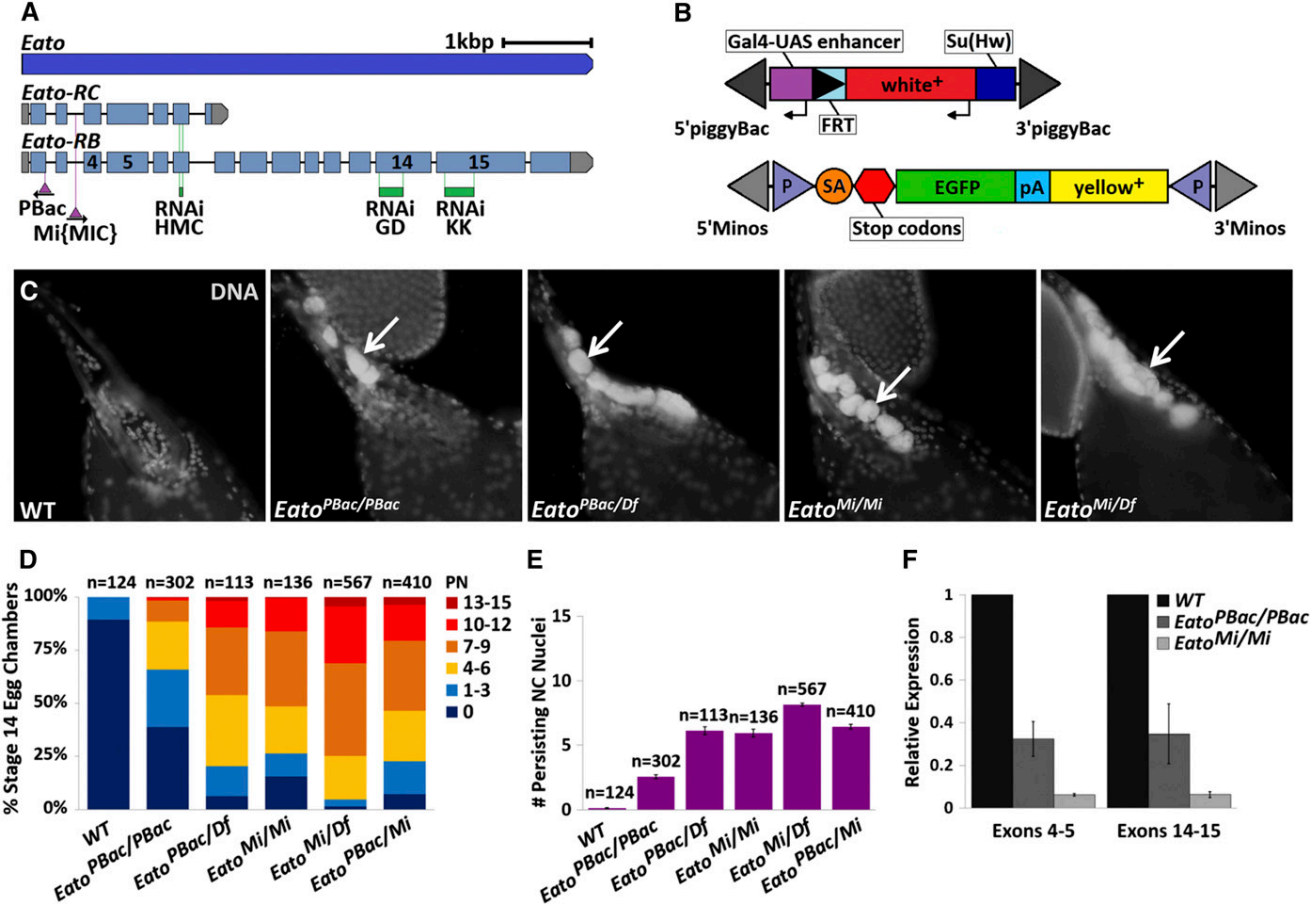
*Eato* mutants have persisting nurse cell (NC) nuclei (PN). (A) Schematic of the *Eato* gene (blue) and the location of transposon insertions and RNA interference (RNAi) target sites. The two mRNA transcripts, *Eato-RC* and *Eato-RB*, are illustrated, showing the translated (light blue) and untranslated regions (gray), the insertion sites of the *PBac* and *Mi{MIC}* transgenic constructs (purple triangles), and the three RNAi target sites (green), with exons 4, 5, 14, and 15 labeled. The *PBac* construct is inserted in the reverse orientation while the *Mi{MIC}* construct is inserted in same orientation as the *Eato* gene (modified from FlyBase). (B) Schematic of the *PBac* and *Mi{MIC}* constructs, modified from Bellen *et al.* (2011) and Venken *et al.* (2011). Thin arrows indicate the direction of transcription. Elements are not drawn to scale. (C) Stage 14 egg chambers, stained with 4′,6-diamidino-2-phenylindole dihydrochloride (DAPI) (white) to label DNA, from wild-type (WT) or *Eato* allelic combinations. WT (*w^1118^*) egg chambers successfully complete programmed cell death and do not have persisting NC nuclei. *Eato^PBac/PBac^* and *Eato^Mi/Mi^* homozygous mutants, and hemizygotes *in trans* to *Df(2L)BSC812* have defects in NC clearance and have PN (arrows). (D) The percentage of egg chambers exhibiting 0 PN, 1–3 PN, 4–6 PN, 7–9 PN, 10–12 PN, and 13–15 PN, of all stage 14 egg chambers quantified per genotype. (E) The average number of persisting NC nuclei in stage 14 egg chambers from each indicated genotype. Error bars indicate ± SEM. (F) *Eato* transcript levels from WT and mutant whole-ovary mRNA samples quantified by quantitative reverse transcriptase polymerase chain reaction using primers flanking the indicated exons. The relative expression is presented after normalization to *Rpl32* as an internal control. Error bars indicate ± SD.

To determine whether *Eato* can act as a functional equivalent for *ced-7/ABCA1* during PCD, we obtained several transposon-induced alleles of *Eato* (Figure 2, A and B) and analyzed them for phenotypes in oogenesis. We focused our initial analysis on late oogenesis because defective clearance is directly quantifiable in late oogenesis compared to midoogenesis. Ovaries from control and *Eato* mutant strains were dissected and then stained with DAPI to label DNA, and the number of NC nuclei persisting in stage 14 egg chambers were counted. The presence of PN indicated a failure in NC clearance.

In wild-type (WT) egg chambers, the 15 germline-derived NCs underwent PCD and were cleared normally, leaving only the mature oocyte by stage 14 (Figure 2C). However, in several *Eato* mutant allelic combinations, we observed significant clearance defects characterized by the presence of PN in stage 14 egg chambers (Figure 2, C–E). Homozygous *Eato^PBac/ PBac^* mutants displayed an average of 2–3 PN, while hemizygous *Eato^PBac/Df^*, *trans*-heterozygous *Eato^PBac/Mi^*, and homozygous *Eato^Mi/Mi^* mutants displayed an average of 6 PN per stage 14 egg chamber. The strongest phenotype was observed in hemizygous *Eato^Mi/Df^* mutants, with stage 14 egg chambers exhibiting an average of 8 PN (Figure 2, D and E). Flies heterozygous for a WT *Eato* allele did not exhibit any notable defects in NC clearance, suggesting that *Eato* is not haploinsufficient.

The *PBac* and *Mi{MIC}* constructs are inserted at the end of the first coding exon and in the third intron of the *Eato* gene, respectively (Figure 2A). The weaker phenotype observed in *Eato^PBac/PBac^* egg chambers relative to the other alleles suggests that the *Eato^PBac^* allele is a weak hypomorph. The *Mi{MIC}* insertion provides a gene trap and a protein trap (Figure 2B) (Venken *et al.* 2011), which in theory should generate a null allele. However, the more severe persisting phenotype observed in hemizygous *Eato^Mi/Df^* mutants compared to homozygous *Eato^Mi/Mi^* mutants suggests that the *Eato^Mi^* allele is a strong hypomorph of *Eato*. RT-qPCR analysis indicated that the *Eato^Mi^* allele is not a null, but is instead a strong hypomorph with a 16.6-fold decrease in transcript levels, while the *Eato^PBac^* allele is a weaker hypomorph with a 3.2-fold decrease in mRNA transcript expression in the ovary relative to WT (*w^1118^)* (Figure 2F).

### Eato is required in the FCs for NC clearance during developmental PCD

We next wanted to discern in which cell type *Eato* function is required to facilitate removal of the NCs. Studies in *C. elegans* showed that CED-7 is required in both the phagocytic cell and the dying cell for efficient engulfment of cell corpses (Wu and Horvitz 1998), while studies in mammals showed that ABCA1 expression in phagocytic cells is sufficient for engulfment (Hamon *et al.* 2000). To determine in which cell type *Eato* acts during engulfment of the NCs, we used tissue-specific drivers to express *Eato* RNAi constructs and knock down *Eato* expression specifically in the FCs or NCs.

**Figure 3:**
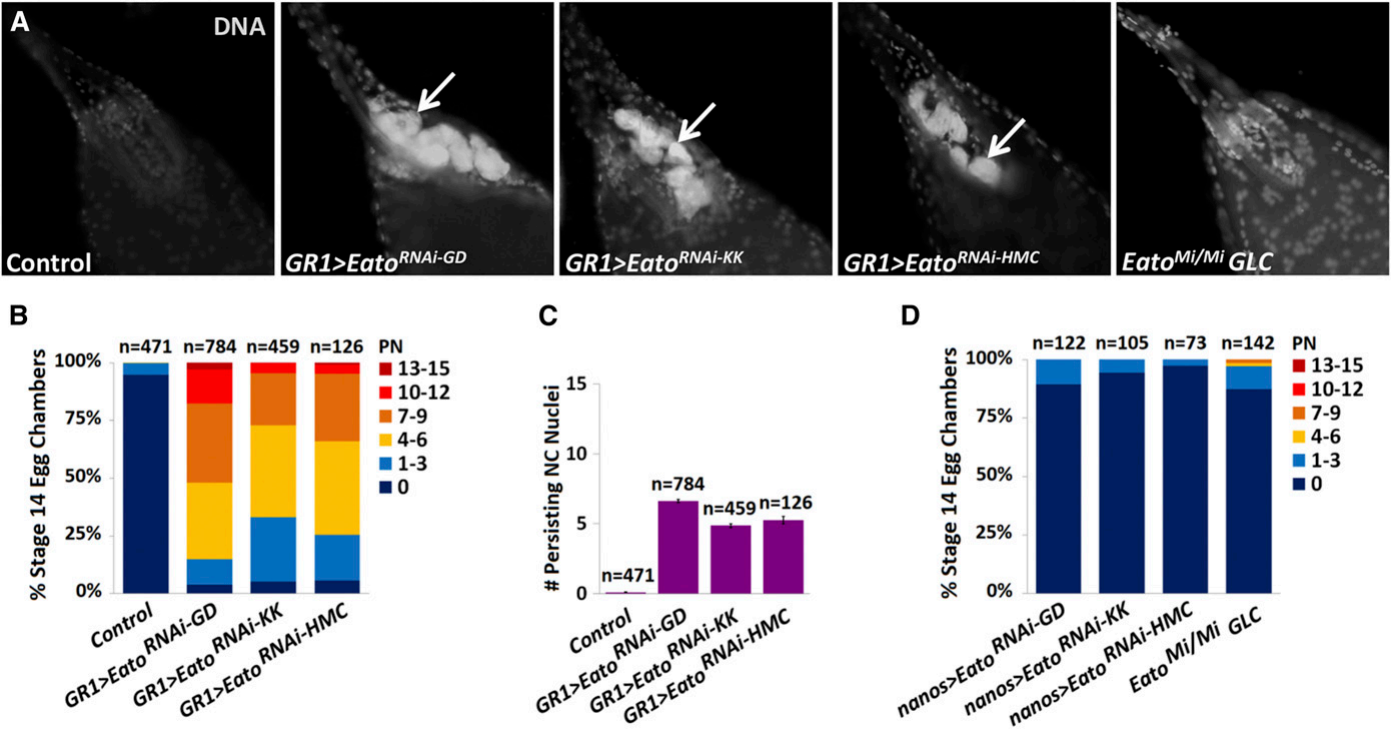
*Eato* is required in the follicle cells (FCs) for nurse cell (NC) clearance. (A) Stage 14 egg chambers, stained with 4′,6-diamidino-2-phenylindole dihydrochloride (DAPI) (white) to label DNA from a sibling control (*UAS-Eato^RNAi^/TM6B*), one of three different *Eato^RNA^*^i^ constructs (*GD1133*, *KK104197*, and *HMC06027*) expressed specifically in the FCs (*GR1-GAL4 > UAS-Eato^RNAi^*), or an *Eato^Mi^* germline clone. Persisting nuclei (PN) (arrows) are observed when *Eato* is knocked down specifically in the FCs but not in the germline clones. (B and D) The percentage of egg chambers exhibiting 0 PN, 1–3 PN, 4–6 PN, 7–9 PN, 10–12 PN, and 13–15 PN, of all stage 14 egg chambers quantified per genotype. (C) The average number of persisting NC nuclei in stage 14 egg chambers from each indicated genotype. Control represents combined *Eato^RNAi^/TM6B* siblings. Error bars indicate ± SEM.

*Eato* knockdown with three different RNAi constructs (Figure 2A) using a FC-specific driver, GR1-GAL4 (*GR1-GAL4 > UAS-Eato^RNAi^*), resulted in stage 14 egg chambers that exhibited PN (Figure 3, A–C). Egg chambers expressing the *GD1133*, *KK104197*, or *HMC06027* RNAi constructs in FCs displayed an average of 7, 5, and 4–5 PN, respectively (Figure 3C). All three RNAi constructs exhibited stronger phenotypes than the *Eato^PBac/PBac^* mutants. The *GD1133* RNAi construct also exhibited a stronger phenotype than the *Eato^Mi/Mi^* mutants, suggesting that PN observed in these mutants can be primarily attributed to loss of *Eato* function in the FCs. Sibling controls (*Eato^RNAi^/TM6B*) containing the RNAi constructs without the driver showed no defects in NC clearance (Figure 3, B and C).

To determine whether *Eato* also acts in dying cells, we knocked down *Eato* specifically in the NCs (*nanos-GAL4 > UAS-Eato^RNAi^*). We did not observe a requirement for *Eato* in the NCs for their clearance. Egg chambers expressing any of the three *Eato^RNAi^* constructs specifically in the NCs did not show any engulfment defects and were able to clear all 15 NCs normally (Figure 3D). While GD and KK RNAi libraries generate long hairpin RNA sequences, which are typically ineffective for RNAi-mediated knockdown in the germline (Ni *et al.* 2011), the *HMC06027* allele encodes a short hairpin RNAi sequence that should competently knock down *Eato* expression in the germline-derived NCs (Ni *et al.* 2011). The lack of persisting NCs in these *Eato* RNAi-expressing stage 14 egg chambers suggests that *Eato* is not required in the dying cells for their clearance. To confirm that *Eato* was not required in the dying NCs, we generated *Eato^Mi^* germline clones. Indeed, stage 14 egg chambers from *Eato^Mi^* germline clones did not show a significant persisting NC phenotype (Figure 3A). However, ∼3% of stage 14 egg chambers exhibited ≥ 4 PN (Figure 3D). We suspect that these egg chambers may have FC clones in addition to germline clones, which can occur in the process of generating germline clones (Peterson and McCall 2013).

### Eato acts in parallel to Ced-12, likely in the same pathway as Drpr

In *C. elegans*, the engulfment mechanism is primarily regulated by two parallel signaling pathways, CED-1/-6/-7 and CED-2/-5/-12 (Ellis *et al.* 1991; Kinchen *et al.* 2005). We have shown that, similar to *C. elegans*, *drpr*, the *ced-1* ortholog, and *Ced-12* act in parallel to regulate clearance of the NCs during *D. melanogaster* oogenesis (Timmons *et al.* 2016). Double knockdowns expressing *Ced-12^RNAi^* and *drpr^RNAi^*, or the null allele *drpr^∆5^*, in the FCs exhibit a significantly more severe persisting NC phenotype compared to either single knockdown alone. In *C. elegans* and mammals, *ced-7/ABCA1* acts in the same pathway as *ced-1/MEGF10*, in parallel to *ced-12/ELMO*. The similarities that we have observed between *Eato* and *ced-7/ABCA1* suggest that *Eato* may act in the same pathway as *drpr* and in parallel to *Ced-12*. To ascertain which pathway *Eato* is involved in during engulfment, we generated *Eato*+*drpr* and *Eato*+*Ced-12* double mutants and analyzed the severity of their engulfment defects in NC clearance.

**Figure 4:**
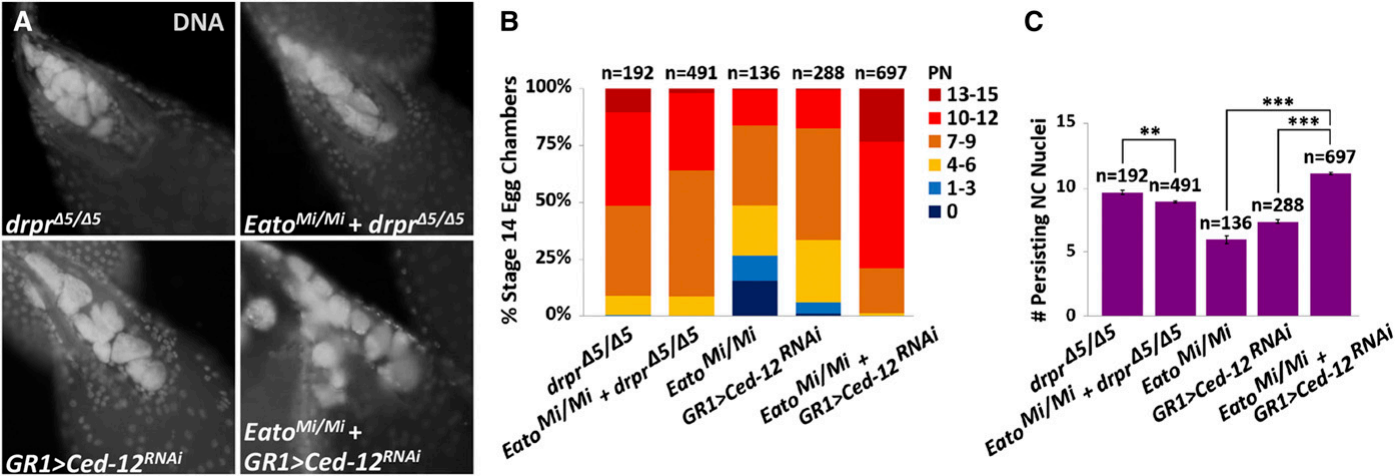
*Eato* acts in the same pathway as *drpr* and in parallel to *Ced-12*. (A) Stage 14 egg chambers, stained with 4′,6-diamidino-2-phenylindole dihydrochloride (DAPI) (white) to label DNA, from *drpr*^Δ^*^5/^*^Δ^*^5^* and *Ced12^RNAi^* expressed specifically in the follicle cells (FCs) (*GR1-GAL4 > UAS-Ced12^RNAi^*), single and double mutants with *Eato^Mi/Mi^*. (B) The percentage of stage 14 egg chambers exhibiting 0 persisting nurse cell (NC) nuclei (PN), 1–3 PN, 4–6 PN, 7–9 PN, 10–12 PN, and 13–15 PN per indicated genotype. (C) The average number of persisting NC nuclei in stage 14 egg chambers from each indicated genotype. Error bars indicate ± SEM. Unpaired *t*-tests were performed: ** *P* < 0.001 and *** *P* < 0.0001.

To determine whether *Eato* acts in the same pathway as *drpr*, we analyzed *Eato^Mi/Mi^*; *drpr*^Δ^*^5/^*^Δ^*^5^* double mutants compared to *drpr*^Δ^*^5/^*^Δ^*^5^* single mutants. We found that the double mutant did not show a stronger phenotype than the single mutant (Figure 4, A–C), and that both *drpr*^Δ^*^5/^*^Δ^*^5^* and the double mutants displayed an average of ∼9 PN (Figure 4C). The similar clearance defects between the single and double mutants indicate that *Eato* acts in the same pathway as *drpr*.

To clarify whether *Eato* acts in parallel to *Ced-12*, we needed to avoid the lethality of *Ced-12* mutants and thus we knocked down *Ced-12* expression only in the phagocytic FCs (*GR1 > Ced-12^RNAi^*). In these *Ced-12* knockdowns, stage 14 egg chambers displayed an average of 7 PN. Impressively, in the *Eato^Mi/Mi^*; *GR1 > Ced-12^RNAi^* double mutants, stage 14 egg chambers displayed an average of 11 PN (Figure 4C). The *Eato^Mi/Mi^*; *GR1 > Ced-12^RNAi^* double mutants displayed a much more severe engulfment defect than either single mutant alone, and a stronger phenotype than the *Eato^Mi/Mi^*; *drpr*^Δ^*^5/^*^Δ^*^5^* double mutants. Moreover, a considerable percentage of these double mutants completely failed to clear any of the NCs (Figure 4B), indicating that the engulfment machinery had been severely impaired. These findings suggest that *Eato* acts in parallel to *Ced-12*, and that its function is important for clearance of the NCs.

### Eato promotes Drpr enrichment and stretch FC membrane extensions surrounding the NCs

During engulfment, CED-1/MEGF10 has been shown to accumulate at the phagocytic cup (Hamon *et al.* 2000; Zhou *et al.* 2001). *In vivo* and *in vitro* studies in *C. elegans* and mouse cell culture, respectively, show that the uniform clustering of CED-1/MEGF10 around cell corpses occurs in a manner dependent on CED-7/ABCA1 activity. To examine whether Eato may function orthologously to CED-7/ABCA1 and be required for Drpr accumulation around the NCs, we analyzed late-stage egg chambers from WT and *Eato^Mi/Mi^* mutants with DAPI to label DNA and anti-Drpr antibody to label Drpr.

**Figure 5:**
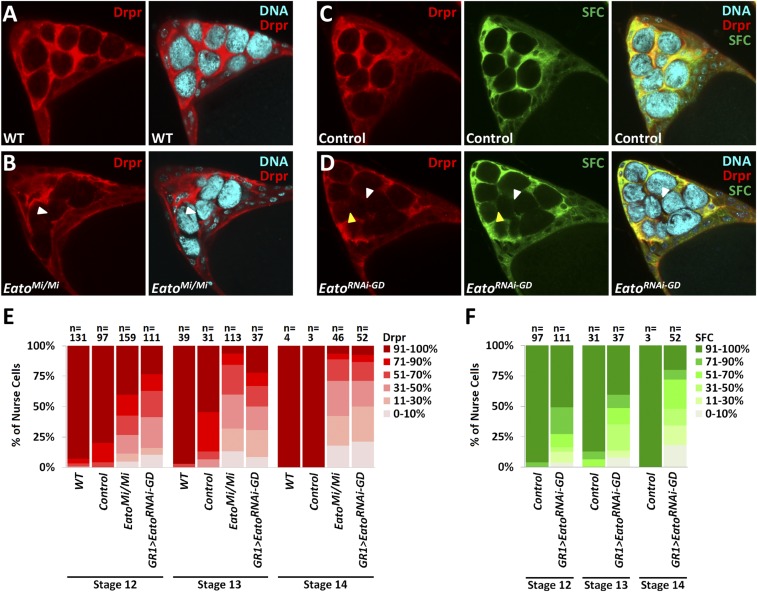
*Eato* is required for Drpr accumulation and follicle cell (FC) membrane extensions around the dying nurse cells (NCs). (A–D) Stage 12 egg chambers, stained with 4′,6-diamidino-2-phenylindole dihydrochloride (DAPI) (cyan), anti-Drpr (red), and expressing green fluorescent protein (GFP) (green) specifically in the SFC membrane (*GR1 > mCD8-GFP*), from wild-type (WT) (*w^1118^*), control (*GR1 > mCD8-GFP*), *Eato^Mi/Mi^* and *Eato* knockdown (*GR1 > mCD8-GFP*, *Eato^RNAi-GD^*) mutants. Areas lacking Drpr staining or GFP expression (white arrowheads) and areas lacking Drpr staining but exhibiting GFP expression (yellow arrowheads) are observed in *Eato* mutants. (E and F) The percentage of NCs exhibiting 0–10, 11–30, 31–50, 51–70, 71–90, and 91–100% Drpr enrichment or GFP in the stretch FC (SFC) membrane around each NC per indicated stage and genotype. In WT and control egg chambers, NCs are normally cleared by stage 14, resulting in an *n* < 10.

In late-stage egg chambers, a subset of anterior FCs, known as the SFCs, associate with the NCs as a squamous epithelium (Wu *et al.* 2008). In WT stage 12–14 egg chambers, Drpr staining becomes enriched in the SFCs and clearly surrounds each NC (Figure 5A) (Timmons *et al.* 2016). However, in *Eato^Mi/Mi^* egg chambers, Drpr staining appeared unevenly enriched and scattered around the NCs. Strikingly, in some areas, Drpr staining was completely absent in the SFCs surrounding the NCs (Figure 5, B and E), indicating that Drpr failed to properly accumulate. These observations suggest a requirement for Eato in Drpr accumulation during NC clearance.

We considered two possible mechanisms that could produce the lack of Drpr staining: either Drpr fails to accumulate at the SFC membrane or the SFC membrane fails to extend and surround the NC. To observe any defects in SFC membrane extensions, we expressed the *mCD8-GFP* transgene, which encodes a membrane-tethered GFP fusion protein, to label FC membranes. In both control (*GR1-GAL4 > UAS-mCD8-GFP*) and *Eato* knockdown egg chambers (*GR1-GAL4 > UAS-mCD8-GFP*, *UAS-Eato^RNAi-GD^*) we observed an overlap of the presence or absence of GFP expression and Drpr staining in the SFCs (Figure 5, C and D). However, in *Eato* knockdown egg chambers, some areas exhibiting GFP did not exhibit Drpr accumulation (Figure 5, D–F). Altogether, these observations indicate a requirement for *Eato* in both SFC membrane extension and Drpr enrichment around the NCs.

### CG1718 may act with Eato in the FCs for NC clearance during developmental PCD

We also examined the engulfment function of *CG1718* in NC clearance during oogenesis. *CG1718* encodes another ABCA protein that is expressed in the ovary, and has been proposed as the *D. melanogaster* homolog of ABCA1 for its role in lipid and cholesterol homeostasis (Bujold *et al.* 2010), and the homolog of *ced-7* in cell clearance (Ziegenfuss 2012; Nainu *et al.* 2017). Additionally, while studies in engulfing glia did not find a role for *CG1718* in neuronal corpse or axonal debris clearance (Ziegenfuss 2012), pan-neuronal-specific knockdown of *CG1718* resulted in synaptic bouton overgrowth at the neuromuscular junction (Ueoka *et al.* 2018). To determine whether *CG1718* plays a role in NC clearance, we quantified PN in *CG1718* knockdowns and mutants.

**Figure 6:**
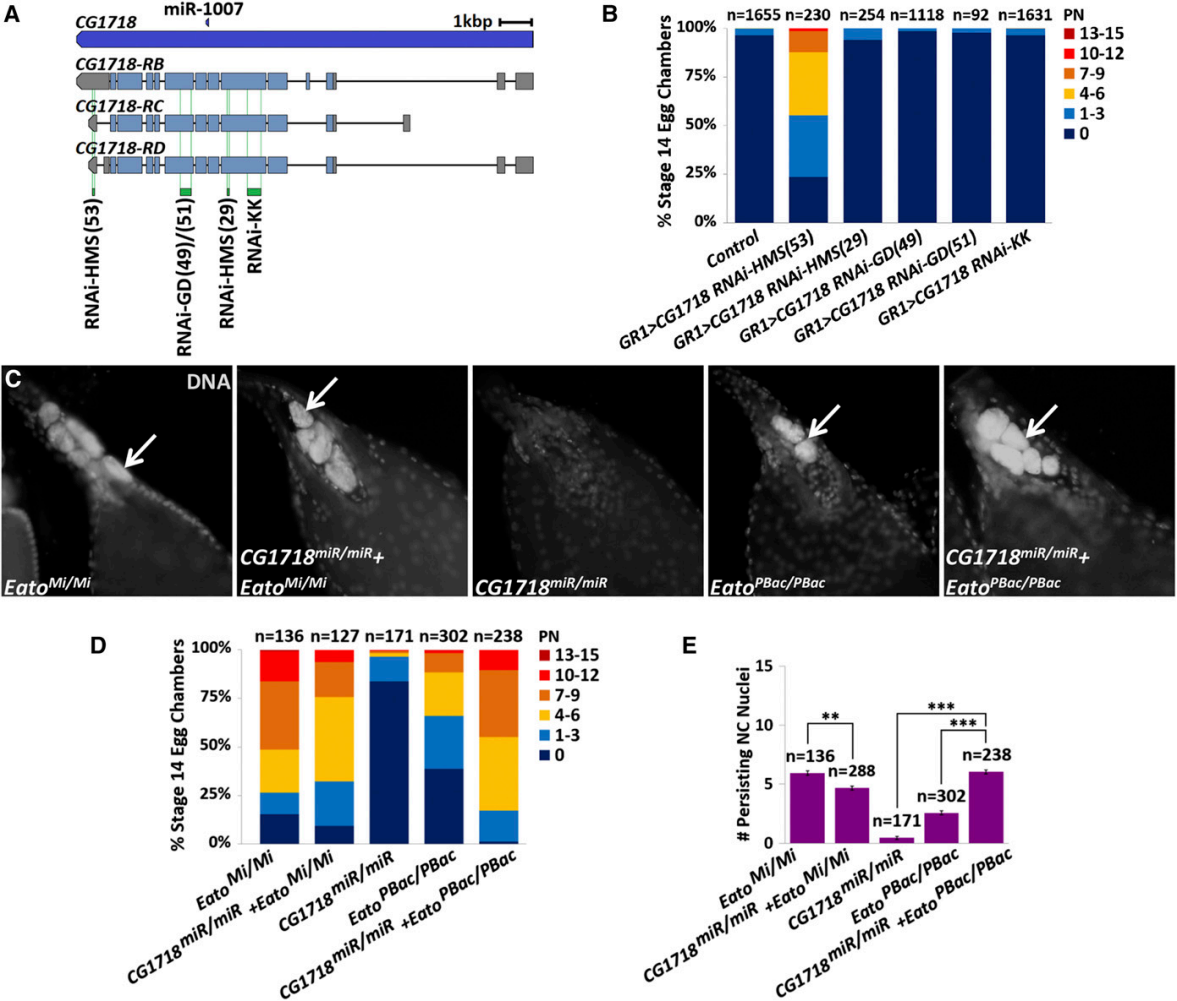
*CG1718* may act with *Eato* in the follicle cells (FCs) for nurse cell (NC) clearance. (A) Schematic of the *CG1718* gene and the three mRNA transcripts, *CG1718-RB*, *CG1718-RC*, and *CG1718-RD*, with the locations of the RNA interference (RNAi) target sites (green). The *miR-1007* gene (dark blue) is also shown (figure modified from FlyBase). (B and D) The percentage of stage 14 egg chambers exhibiting 0 persisting NC nuclei (PN), 1–3 PN, 4–6 PN, 7–9 PN, 10–12 PN, and 13–15 PN per indicated genotype. (C) Stage 14 egg chambers, stained with 4′,6-diamidino-2-phenylindole dihydrochloride (DAPI) (white) to label DNA, from *Eato^Mi/Mi^* mutants, *Eato^PBac/ PBac^* mutants, *CG1718^miR/miR^* mutants, or double mutant combinations. PN are shown (arrows). (E) The average number of PN in stage 14 egg chambers from each indicated genotype. Error bars indicate ± SEM. Unpaired *t*-tests were performed: ** *P* < 0.001 and *** *P* < 0.0001.

Egg chambers expressing one of five *CG1718^RNAi^* constructs (Figure 6A) specifically in the NCs (*nanos > CG1718^RNAi^*) did not exhibit any engulfment defects (data not shown). Intriguingly, while egg chambers expressing any one of four *CG1718^RNAi^* constructs specifically in the FCs (*GR1 > CG1718^RNAi^*) did not exhibit any engulfment defects, expression of the *HMS01821* RNAi construct specifically in the FCs resulted in moderate engulfment defects as 45% of these stage 14 egg chambers exhibited ≥ 4 PN (Figure 6B). Unique from the other *CG1718* RNAi constructs, the *HMS01821* RNAi sequence targets the 3′ untranslated region (3′UTR) of *CG1718* transcripts (Figure 6A).

We also examined a *miR-1007* deletion strain (*CG1718^miR/miR^*), in which the expression of *CG1718* was reported to be reduced (Chen *et al.* 2014). The *miR-1007* gene is nestled in one of the introns of *CG1718* (Figure 6A), and a systematic study of *Drosophila* microRNA (miR) functions previously generated the *CG1718^miR/miR^* strain. Stage 14 egg chambers from these mutants did not exhibit any pronounced NC clearance defects (Figure 6, C–E).

ABC transporters of the ABCA family share a common function in lipid transport (Vasiliou *et al.* 2009; Quazi and Molday 2011), and thus we wondered whether *CG1718* and *Eato* could provide compensatory or redundant roles for each other in NC clearance. We generated *CG1718*+*Eato* double mutants carrying homozygous *CG1718^miR/miR^* and *Eato^Mi/Mi^*, or *Eato^PBac/PBac^*, hypomorphic alleles. Interestingly, egg chambers from *CG1718^miR/miR^*; *Eato^Mi/Mi^* double mutants exhibited a less severe clearance defect than *Eato^Mi/Mi^* mutants alone, with an average of 4–5 PN. However, egg chambers from *CG1718^miR/^^miR^*; *Eato^PBac/PBac^* double mutants exhibited a much more severe PN phenotype than either single mutant alone, with an average of 6 PN per stage 14 egg chamber (Figure 6E). While the less severe phenotype observed in *CG1718^miR/miR^;Eato^Mi/Mi^* double mutants remains bewildering, the much more severe phenotype observed in *CG1718^miR/miR^*; *Eato^PBac/PBac^* double mutants suggests a functional relationship between the two ABCA transporters. In mammals, ABCA1 and ABCA7 have been demonstrated to provide redundant functions (Wang *et al.* 2003; Abe-Dohmae *et al.* 2004; Kim *et al.* 2005). Perhaps in *D. melanogaster*, *CG1718* could provide a compensatory or redundant function for *Eato*.

### Eato is required in the FCs for engulfment of the germline during stress-induced PCD

To determine if *Eato* is required for clearance in other forms of cell death, we investigated whether *Eato* plays a role during starvation-induced PCD in midoogenesis. In response to starvation, NCs die via apoptosis during midoogenesis (Jenkins *et al.* 2013) and are engulfed by surrounding epithelial FCs. The cell death of the NCs in midoogenesis proceeds through five morphologically distinct phases defined by changes in the NC chromatin (Etchegaray *et al.* 2012). In phase 0 healthy egg chambers, the 15 NCs exhibit dispersed chromatin; in phase 1 dying egg chambers, the NC chromatin becomes disordered and begins to condense; by phase 3 the NC chromatin becomes highly condensed in single large balls and the FCs appear enlarged; and by phase 5 the FCs have phagocytosed the germline material and constitute almost the entire egg chamber.

From our studies in late oogenesis, we selected the *Eato* mutants that exhibited the most severe phenotypes and looked for engulfment defects in midoogenesis. We selected *Eato^Mi/Df^* and FC knockdown *Eato^RNAi-GD^* mutants and starved the adults for a 16–20 hr period to induce PCD in midstage egg chambers. Subsequently, we dissected and stained the ovaries with DAPI to label DNA, anti-Dlg to label FC membranes, and anti-cleaved Dcp-1 to label active caspases and apoptotic germline debris. We analyzed dying egg chambers from each phase, sorted by the changes in NC chromatin, and compared the progression of engulfment to control dying egg chambers.

**Figure 7:**
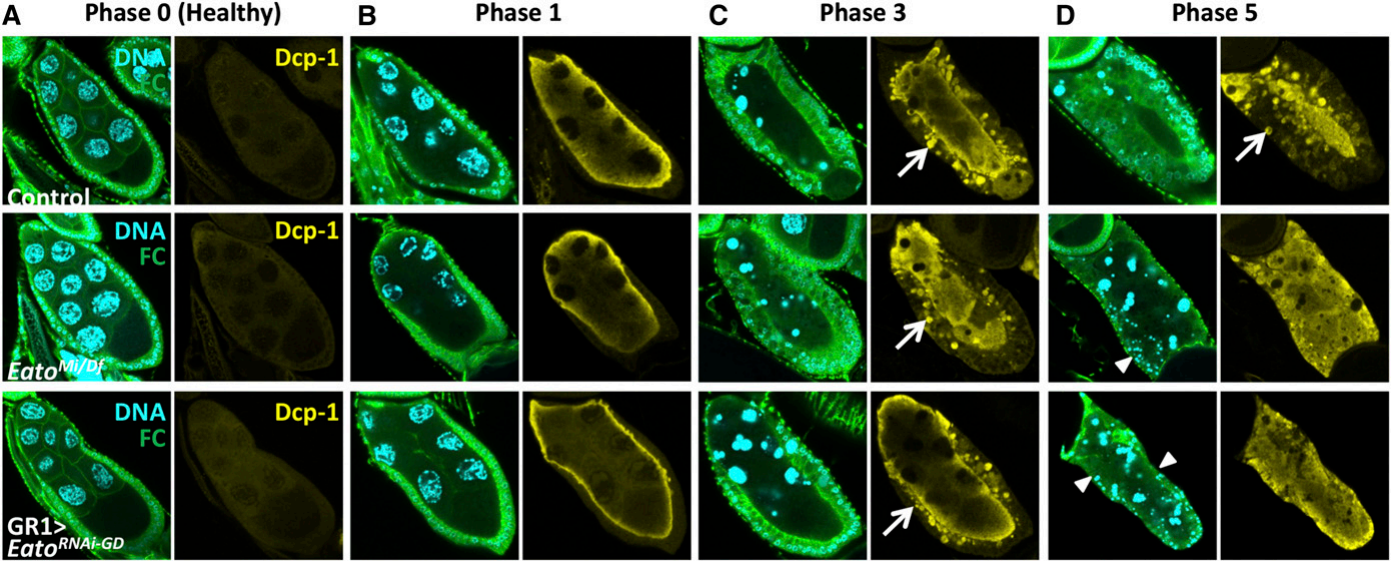
*Eato* is required in the follicle cells (FCs) for engulfment of nurse cells (NCs) during starvation-induced programmed cell death. Egg chambers stained with anti-Dlg antibody (green) to label FC membranes, anti-cleaved Dcp-1 antibody (yellow) to label active caspases and apoptotic germline debris, and 4′,6-diamidino-2-phenylindole dihydrochloride (DAPI) (cyan) to label DNA. (A) Phase 0 healthy egg chambers. (B) Phase 1 dying egg chambers of *Eato^Mi/Df^* mutants, or when *Eato^RNAi-GD1133^* is expressed specifically in the FCs (*GR1-GAL4 > UAS-Eato^RNAi-GD1133^*), resemble the control (*UAS-Eato^RNAi-GD1133^/TM6B*) dying egg chambers. (C) Phase 3 dying egg chambers of *Eato* mutants exhibit enlarged FCs; however, they show fewer Dcp-1-positive vesicles inside the FCs (arrows). (D) Phase 5 dying egg chambers of *Eato* mutants show severe engulfment defects with completely unengulfed germline and pyknotic FC nuclei (arrowheads) without membrane markers.

Similar to the results in late oogenesis, *Eato* mutants exhibited engulfment defects and showed FCs that failed to enlarge normally but also died prematurely, leaving behind egg chambers with unengulfed germline debris. In both *Eato^Mi/Df^* and *GR1 > Eato^RNAi-GD^* mutants, healthy phase 0 egg chambers resembled the control, suggesting that *Eato* mutant egg chambers develop normally through midoogenesis (Figure 7A). Phase 1 dying egg chambers also resembled the control, appearing to activate caspases and initiate PCD normally (Figure 7B). While phase 3 dying egg chambers exhibited enlarged FCs, they appeared to show fewer engulfed Dcp-1-positive vesicles present inside the FCs compared to control (Figure 7C). By phase 5, *Eato* mutant dying egg chambers exhibited severe engulfment defects and FC death. In phase 5 dying egg chambers, the majority of the FCs exhibited pyknotic nuclei without any membrane markers or disappeared, leaving behind egg chambers with completely unengulfed germline material (Figure 7D), resembling *drpr* mutants (Etchegaray *et al.* 2012). Many of the dying egg chambers found in the ovaries of *Eato* mutants were these phase 5 egg chambers, indicating a pronounced defect in removal of these egg chambers. These observations signify severe impairments in the engulfment machinery in the later steps of engulfment, and illustrate an important role for *Eato* in the FCs for engulfment of the germline debris during midstage apoptotic death.

We did not observe any obvious engulfment defects in dying egg chambers when *Eato^RNAi-GD^* or *Eato^RNAi-HMC^* was expressed only in the germline (data not shown), suggesting that, as in late oogenesis, *Eato* is primarily required in the phagocytic FCs during engulfment of the dying germline material.

## Discussion

Here, we report the characterization of *Eato*, which encodes an ABC transporter that is structurally and functionally similar to *ced-7/ABCA1*. Like *ced-7/ABCA1*, *Eato* encodes an ABCA type transporter and presents substantial sequence similarity and identity to that of CED-7 and ABCA1. Moreover, our genetic analyses identify a role for *Eato* in cell clearance during PCD, demonstrating functional conservation. To our knowledge, prior to this study there have been no reports of a *D. melanogaster* functional equivalent for CED-7/ABCA1.

Using the *D. melanogaster* ovary as an *in vivo* model system, we observed cell clearance defects in *Eato* mutants in both developmental PCD, which proceeds via phagoptosis (Timmons *et al.* 2016), and starvation-induced PCD, which proceeds via apoptosis (Jenkins *et al.* 2013). Unlike in *C. elegans*, where CED-7 was reported to be required both in the dying cell and the phagocytic cell for corpse clearance (Wu and Horvitz 1998), our investigation indicates a requirement for *Eato* only in the phagocytic FCs and not in the dying NCs. Genetically knocking down *Eato* specifically in the phagocytic FCs resulted in engulfment defects, while knocking down *Eato* expression specifically in the germline did not affect clearance. These findings demonstrate a conserved role for *Eato* in two distinct PCD modalities, specifically in the phagocytic cells for cell clearance.

In *C. elegans* and mammals, *ced-7/ABCA1* acts in the same pathway as *ced-1/MEGF10*, in parallel to *ced-12/ELMO* (Ellis *et al.* 1991; Kinchen *et al.* 2005; Mangahas and Zhou 2005). Correspondingly, our double mutant analyses of *Eato^Mi/Mi^* with *drpr*^Δ^*^5/^*^Δ^*^5^*or *Ced-12^RNAi^* show that *Eato* acts in the same pathway as *drpr* and in parallel to *Ced-12*, strongly suggesting that *Eato* provides a *ced-7/ABCA1*-like role in this conserved engulfment mechanism. Indeed, we observed a requirement for *Eato* in Drpr enrichment around the NCs, similar to the requirement for CED-7/ABCA1 in CED-1/MEGF10 clustering at the phagocytic cup (Zhou *et al.* 2001; Hamon *et al.* 2006).

Multiple investigations in *C. elegans* and mammals have reported a role for CED-7/ABCA1 in lipid transport (Hamon *et al.* 2000; Wang *et al.* 2010; Mapes *et al.* 2012). Importantly, CED-7 has been implicated in intracellular vesicle delivery to the phagocytic cup to provide membrane material (Yu *et al.* 2006). Our investigation showed that in *Eato* mutants, phagocytic membrane extensions around the NCs are disrupted. In both late-stage and dying midstage egg chambers from *Eato* mutants, the FCs fail to extend and complete engulfment of the germline debris. Thus, we speculate that Eato may function as a lipid transporter to deliver vesicles containing membrane material and other proteins, such as Drpr, to the growing FC membrane. This would explain the defects observed in SFC membrane extensions and Drpr accumulation in late oogenesis, and the FC enlargement and germline uptake in midoogenesis in *Eato* mutants.

Transporters of the ABCA family are commonly involved in lipid trafficking, though the specific substrates transported by CED-7/ABCA1 remain to be identified. Cell culture experiments have suggested that ABCA1 may act as a translocase to translocate the “eat-me signal” PtdSer from the inner leaflet to the outer leaflet (Hamon *et al.* 2000; Smith *et al.* 2002; Albrecht *et al.* 2005). However, *in vivo* PtdSer exposure on the surface of dying cells does not require CED-7 in *C. elegans*. Instead, CED-7 was shown to be required for the transfer of PtdSer-containing vesicles from the surface of dying cells to the surface of phagocytic cells (Mapes *et al.* 2012), suggesting that CED-7 can efflux PtdSer and potentially other phospholipids. In the context of HDL formation, ABCA1 has also been speculated to preferentially transport phosphatidylcholine (Takahashi *et al.* 2006). Our identification of Eato provides another system to elucidate the transport activities of this unique class of proteins in engulfment.

In mammals, ABCA1 and ABCA7 have been observed to provide homologous functions, especially in lipid homeostasis (Wang *et al.* 2003; Abe-Dohmae *et al.* 2004; Hamon *et al.* 2006). Like ABCA1, ABCA7 has been demonstrated to mediate phospholipid and cholesterol release to form HDLs, and even compensate for ABCA1 in certain conditions (Wang *et al.* 2003; Abe-Dohmae *et al.* 2004; Kim *et al.* 2005). Similarly, we observed a role for another *ABCA*-encoding gene, *CG1718*, in NC clearance. Most noticeably, in an *Eato^PBac/PBac^* hypomorphic background, *CG1718* may provide a compensatory or redundant function for *Eato* during NC clearance. Since both genes encode ABC transporters of the same family, which are known to share a functional relationship in lipid trafficking, the proteins may be able to provide similar if not the same functions. Thus, as in the case of ABCA1 and ABCA7, *CG1718* may share redundant functions or possibly provide compensatory mechanisms in the absence of *Eato*.

*CG1718* pan-neuronal knockdown flies were recently established as a model for autism spectrum disorder. These flies exhibited behavioral characteristics similar to those observed in human autism spectrum disorder patients, and showed excessive synaptic satellite bouton outgrowths (Ueoka *et al.* 2018), similar to those in *Fmr1* mutants (Zhang *et al.* 2001). The *Fmr1* gene has been reported to play a role in glial phagocytosis of neuronal and axonal debris, and in hemocyte phagocytosis of bacteria (Logan 2017; O’Connor *et al.* 2017), and thus, by analogy, *CG1718* may similarly play a phagocytic role in cell clearance, though likely not in engulfing glia (Ziegenfuss 2012).

We also looked for a role for *Eato* in glial phagocytosis of neuronal debris. *Eato^Mi/Mi^* mutants did not show any accumulation of uncleared neuronal corpses, suggesting that *Eato* does not play a prominent role during corpse clearance in the brain. We speculate that perhaps there is another *ABCA* gene that provides a *ced-7-/ABCA1*-like role during PCD events in engulfing glia, perhaps *CG34120*, which appears to be the most appreciably expressed ABCA transporter in the head (FlyBase).

*Eato* may also be involved in salivary gland clearance during development. *Eato* is expressed at high levels in the salivary glands (FlyBase), which undergo autophagic cell death and are cleared during larval development. *E93* mutants, which exhibit persisting salivary glands (Lee and Baehrecke 2001), show decreased expression of *Eato* (Dutta 2008). Additionally, Drpr was also found to be enriched in the salivary glands and to be required for salivary gland clearance (McPhee and Baehrecke 2010). As our study indicates a relationship between *drpr* and *Eato*, it seems likely that *Eato* may have a role in degradation and clearance of the salivary glands.

In conclusion, our findings provide insight into the molecular activities that occur during engulfment in PCD, with specific attention to the role of ABCA transporters. We have identified *Eato*, a *ced-7/ABCA1*-like ABCA transporter gene that is required during engulfment in the *D. melanogaster* ovary. To our knowledge, this is the first report of a role for ABCA transporters in PCD in *Drosophila*. Further characterization of this *ced-7/ABCA1* ortholog in *D. melanogaster* will help elucidate the functions and mechanisms of this unique class of transporters during PCD.
